# An Intelligent Trial Eligibility Screening Tool Using Natural Language Processing With a Block-Based Visual Programming Interface: Development and Usability Study

**DOI:** 10.2196/80072

**Published:** 2025-12-11

**Authors:** Ya-Han Hu, Yi-Ying Cheng, Chung-Ching Lan, Yu-Hsiang Su, Sheng-Feng Sung

**Affiliations:** 1 Department of Information Management, National Central University, Taoyuan, Taiwan; 2 Asian Institute for Impact Measurement and Management, National Central University, Taoyuan, Taiwan; 3 Division of Neurology, Department of Internal Medicine, Ditmanson Medical Foundation Chia-Yi Christian Hospital, 539 Zhongxiao Road, East District, Chiayi City, 60002, Taiwan, +886-5-2765041 ext 8655; 4 Department of Nursing, Fooyin University, Kaohsiung, Taiwan

**Keywords:** block-based visual programming, clinical decision support, clinical trials, electronic medical records, eligibility screening, natural language processing, patient safety

## Abstract

**Background:**

Clinical trial eligibility screening using electronic medical records (EMRs) is challenging due to the complexity of patient data and the varied clinical terminologies. Manual screening is time-consuming, requires specialized knowledge, and can lead to inconsistent participant selection, potentially compromising patient safety and research outcomes. This is critical in time-sensitive conditions like acute ischemic stroke. While computerized clinical decision support tools offer solutions, most require software engineering expertise to update, limiting their practical utility when eligibility criteria change.

**Objective:**

We developed and evaluated the intelligent trial eligibility screening tool (iTEST), which combines natural language processing with a block-based visual programming interface designed to enable clinicians to create and modify eligibility screening rules independently. In this study, we assessed iTEST’s rule evaluation module using pre-configured rules and compared its effectiveness with that of standard EMR interfaces.

**Methods:**

We conducted an experiment at a tertiary teaching hospital in Taiwan with 12 clinicians using a 2-period crossover design. The clinicians assessed the eligibility of 4 patients with stroke for 2 clinical trials using both standard EMR and iTEST in a counterbalanced order, resulting in 48 evaluation scenarios. The iTEST comprised a rule authoring module using Google Blockly and a rule evaluation module utilizing MetaMap Lite for extracting medical concepts from unstructured EMR documents and structured laboratory data. Primary outcomes included accuracy in determining eligibility. Secondary outcomes measured task completion time, cognitive workload using the National Aeronautics and Space Administration Task Load Index scale (range 0‐100, with lower scores indicating a lower cognitive workload), and system usability through the system usability scale (range: 0‐100, with higher scores indicating higher system usability).

**Results:**

The iTEST significantly improved accuracy scores (from 0.91 to 1.00, *P*<.001) and reduced completion time (from 3.18 to 2.44 min, *P*=.004) compared to the standard EMR interface. Users reported lower cognitive workload (National Aeronautics and Space Administration Task Load Index scale, 39.7 vs 62.8, *P*=.02) and higher system usability scale scores (71.3 vs 46.3, *P*=.01) with the iTEST. Particularly notable improvements in perceived cognitive workload were observed in temporal demand, effort, and frustration levels.

**Conclusions:**

The iTEST demonstrated superior performance in clinical trial eligibility screening, delivering improved accuracy, reduced completion time, lower cognitive workload, and better usability when evaluating preconfigured eligibility rules. The improved accuracy is critical for patient safety, as the misidentification of eligibility criteria could expose patients to inappropriate treatments or exclude them from beneficial trials. The adaptability and ability of the iTEST to process both structured and unstructured data make it valuable for time-sensitive scenarios and evolving research protocols. Future research should evaluate clinicians’ ability to create and modify eligibility rules using the block-based authoring interface, as well as assess the iTEST across diverse types of clinical trials and health care settings.

## Introduction

Clinical trials follow strict criteria for participant selection, using documented medical records as essential information sources. Over time, electronic medical records (EMRs) have evolved into comprehensive data repositories, enabling health care providers to track information systematically [1-3]. However, the increased volume and complexity of EMRs offer a detailed view of patient histories while posing challenges for researchers analyzing this wealth of data.

The complexity of EMRs demands extensive time and effort from researchers to analyze documents. Addressing EMR-related cognitive overload and burnout is now critical [4]. Researchers face tight deadlines, especially in clinical trials for time-sensitive conditions like stroke or brain injury. The phrase “time is brain” underscores the urgency for immediate therapy post-onset [5,6]. Therefore, researchers must rapidly verify inclusion and exclusion criteria [7,8]. Accurately extracting relevant information from EMRs is also essential for participant eligibility; violations could compromise trial validity and harm participants. This heavy workload increases mental stress and risks judgment errors among clinical researchers.

To address these challenges, using computerized clinical decision support tools in trial eligibility screening offers a promising solution [9]. These systems can efficiently process EMRs, enabling rapid candidate screening. However, EMRs contain a significant amount of unstructured narrative text, with about 80% being narrative—a format clinicians prefer for patient information [10]. These unstructured data complicate medical concept extraction automation. The widespread use of synonyms, acronyms, and abbreviations in clinical documentation further complicates matters, making conventional natural language processing (NLP) techniques often inadequate and necessitating specialized medical NLP tools.

Our previous study developed an NLP-enhanced task-specific EMR interface that presented relevant medical concepts to clinicians through highlighted documents for determining intravenous thrombolysis eligibility criteria [11]. The results showed that this EMR interface improved decision-making in stroke therapy. However, creating entirely new systems with specific rules for each clinical problem remains challenging. Clinical trial or therapy eligibility criteria often evolve as new evidence emerges [12]. Current approaches require software engineers to update rules or build new systems whenever clinical guidelines or criteria change, raising costs [13]. Therefore, there is considerable room for improving the design of such tools.

This study aimed to develop an intelligent trial eligibility screening tool (iTEST) adaptable to various medical scenarios, particularly where eligibility criteria frequently change. By identifying common patterns in eligibility criteria and using a block-based visual programming interface, our tool enables clinicians to create computerized eligibility rules independently, without requiring software engineering expertise. We conducted experiments to evaluate the tool’s impact on users’ accuracy, efficiency, and cognitive workload during eligibility determination.

## Methods

### Study Setting

This study was conducted at Ditmanson Medical Foundation Chia-Yi Christian Hospital, a 1000-bed tertiary teaching hospital in southern Taiwan, which has a certified comprehensive stroke center managing approximately 650 stroke admissions annually. We evaluated 2 sets of eligibility criteria: the first set for intravenous thrombolysis, adapted from the National Institute of Neurological Disorders and Stroke (NINDS) recombinant tissue-plasminogen activator (rt-PA) trial [14], which examined the efficacy of tissue plasminogen activator for acute ischemic stroke, and the second set from the Librexia STROKE trial [15], examining milvexian’s safety and efficacy in preventing recurrent cerebrovascular events post-acute ischemic stroke or high-risk transient ischemic attack.

### Ethical Considerations

The study protocol received formal approval from the Institutional Review Board of Ditmanson Medical Foundation Chia-Yi Christian Hospital (2022102). A unique study identification number replaced patient identifiers to ensure confidentiality. Informed consent was thus exempted. Participants were compensated NTD 3000 (≈USD 96) for their time and participation.

### Intelligent Trial Eligibility Screening Tool

**Figure 1.**
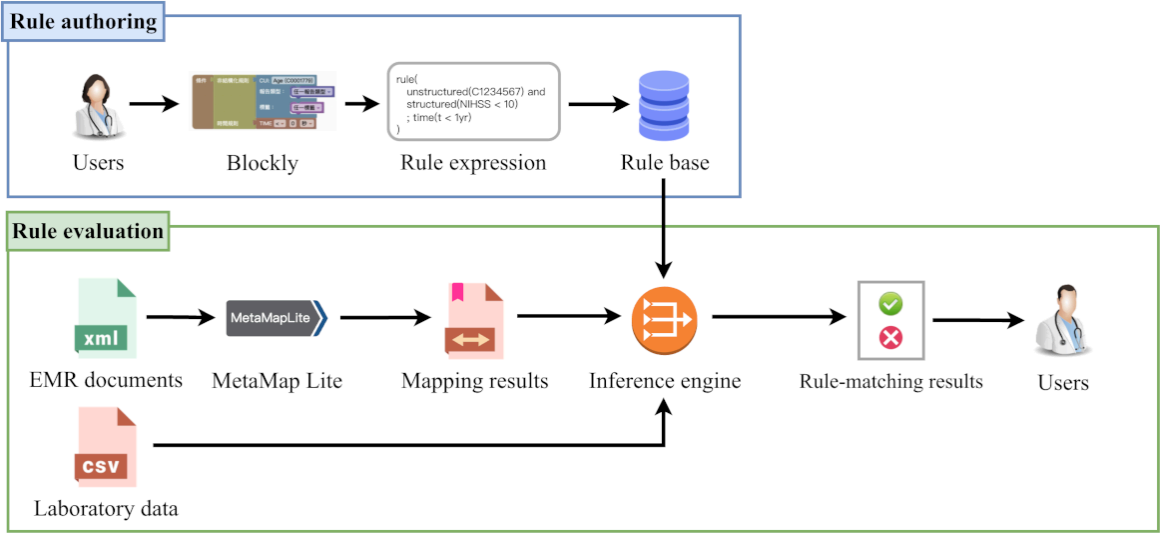
Overview of the intelligent trial eligibility screening tool (iTEST), which comprises a rule authoring module (upper part) and a rule evaluation module (lower part). EMR: electronic medical record.

Figure 1 illustrates the overview of the iTEST, which has 2 main components: the rule authoring and evaluation modules. In the rule authoring module, users convert narrative eligibility criteria into Blockly blocks [16], which are parsed into customized rule expressions and stored in the rule base. The rule evaluation module extracts structured laboratory data from EMRs and maps medical concepts from unstructured EMR documents using MetaMap Lite [17]. The inference engine then determines eligibility for each criterion by matching the extracted information to the rule base, and the tool presents users with rule-matching results.

**Figure 2.**
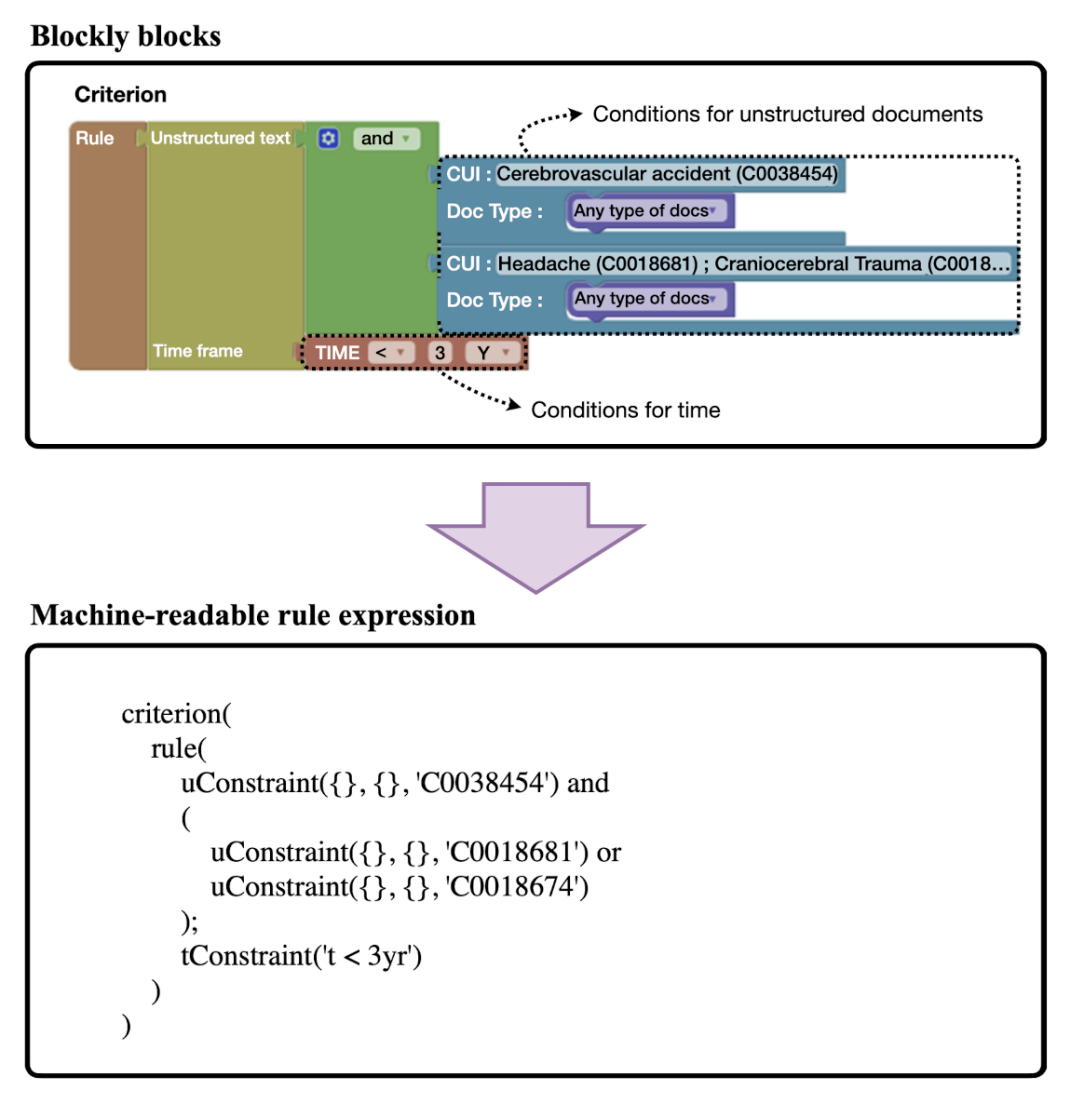
An example showing how Blockly blocks are converted into machine-readable rule expressions.

The rule authoring module (upper part of Figure 1) creates, edits, and versions the eligibility criteria checklist using a block-based visual programming interface powered by Blockly (version 10.4.3; Google) [16]. This method, commonly used in programming education, provides visual cues and drag-and-drop functionality while avoiding invalid block combinations. Our study developed customized rule expressions that represent medical concepts and laboratory items in a machine-readable format. Users can easily convert human-written checklist criteria into rule expressions by creating criterion blocks. For numerical laboratory data, criterion blocks allow users to input laboratory items to define conditions (eg, “Platelets <100,000”). For unstructured documents, criterion blocks enable users to specify multiple medical concepts and select EMR document types. Time conditions can be added by using temporal blocks that perform date calculations on EMR timestamp fields (Figure 2), while logical operators such as AND can combine blocks, which execute separate queries across EMR documents, to express complex criteria like “Having a headache or head injury within three years” or “diabetes with prior stroke in the past.” The module translates these blocks into machine-readable rule expressions, enhancing the readability of eligibility criteria and making it easier for nonprogrammers to understand. This module thus enables clinicians to develop complex eligibility rules without requiring programming skills.

The rule evaluation module (lower part of Figure 1) processes 2 data types. It reads numerical laboratory data in tabular format and uses MetaMap Lite (3.6.2rc8 binary only version; U.S. National Library of Medicine) to extract medical concepts from unstructured EMR documents. MetaMap Lite [17], a faster version of MetaMap [18], maps concepts using the Unified Medical Language System Metathesaurus and assigns Unified Medical Language System concept unique identifiers to annotated concepts [17]. It generates a list of concepts from EMR documents, discarding irrelevant ones based on semantic type settings. Additionally, it addresses challenges like complex concepts with multiple synonyms and potential misinterpretation of word context variations.

As an internal validation, we evaluated MetaMap Lite’s ability to identify medical concepts related to the eligibility criteria examined in this study. We randomly selected 61 documents from 20 patients in a validation dataset separate from the one used for rule development. Two independent expert clinicians manually reviewed and annotated each document to establish a gold-standard reference, resolving disagreements through discussion and consensus. Precision and recall were calculated by comparing MetaMap Lite’s automated extractions with the gold-standard annotations for each eligibility concept. Table S1 in Multimedia Appendix 1 displays the results, showing high precision and recall for most of the related concepts.

**Figure 3.**
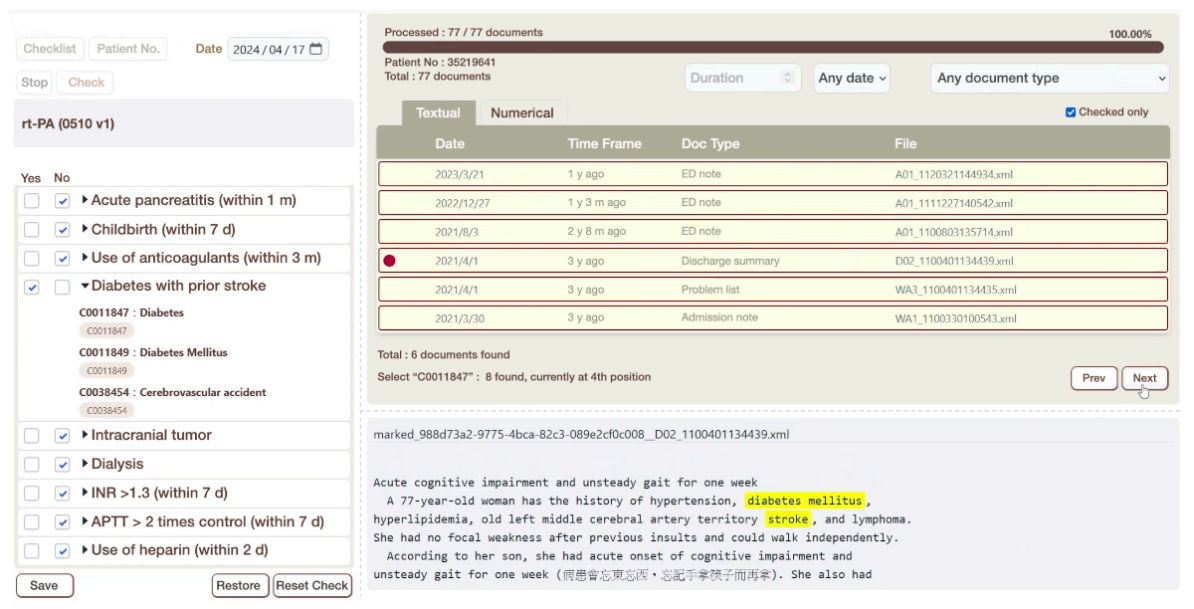
The intelligent trial eligibility screening tool (iTEST) interface. The left panel displays a trial eligibility criteria checklist, with each criterion marked yes or no based on the patient’s data. The upper right panel features 2 tabs: one for clinical documents and one for numerical laboratory data. A red dot marks documents with relevant information. The lower right panel shows the content of the selected clinical document, highlighting key medical concepts for easy reference.

The inference engine analyzes the extracted information against the rule base to evaluate eligibility for each criterion. A web-based interface displays the rule-matching results, allowing users to review the eligibility criteria checklist with links to annotated EMR documents, highlighting key concepts for review (Figure 3).

### User Experiment

We conducted a user experiment to assess users’ accuracy, efficiency, and perceived workload in determining eligibility. A total of 12 clinicians participated in the study, using either a standard EMR interface or the iTEST on a desktop computer to complete eligibility criteria checklists for the NINDS rt-PA and Librexia STROKE trials. Participation was voluntary and compensated.

**Figure 4.**
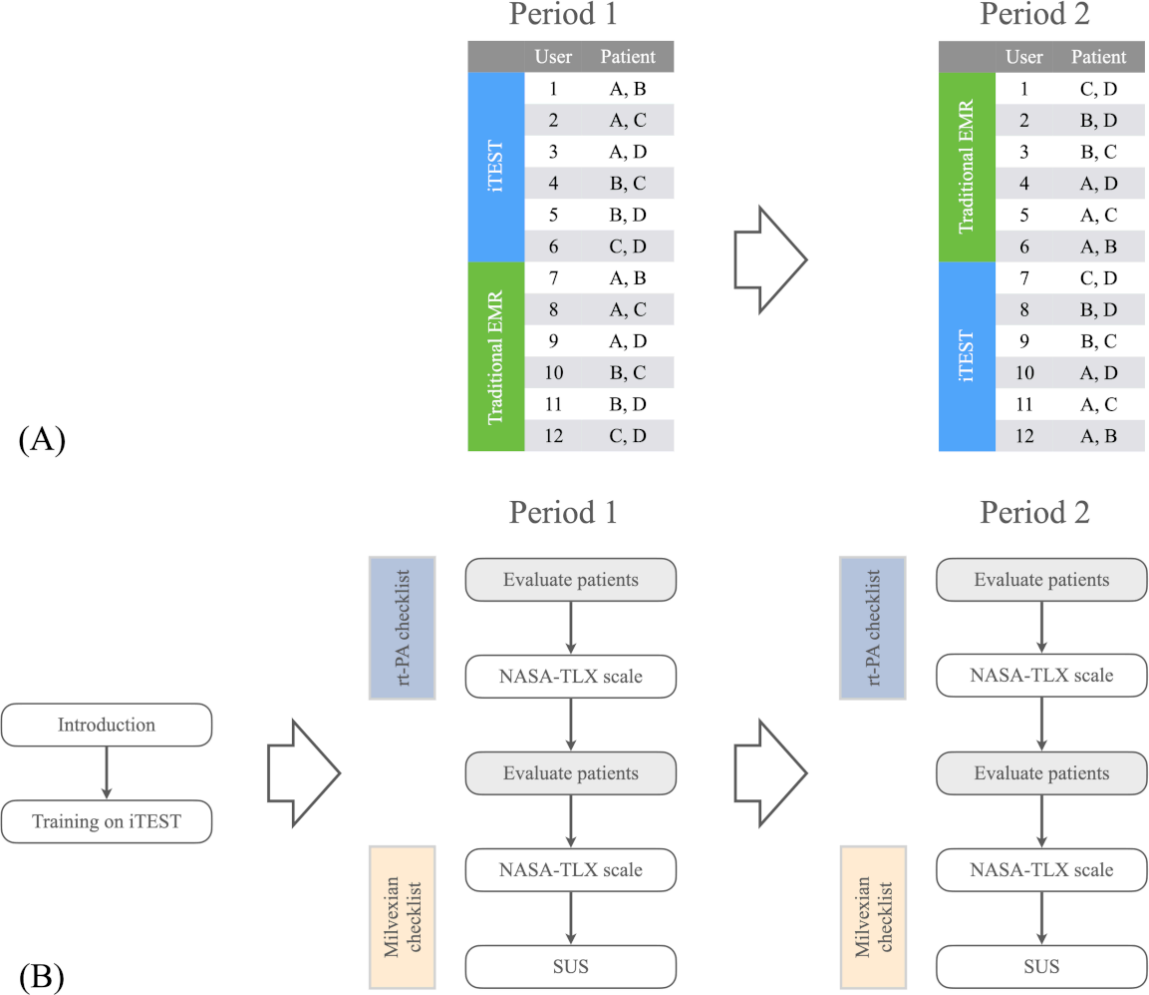
A 2-period crossover design for the user experiment (A). Procedures of the user experiment (B). EMR: electronic medical record; NASA-TLX: National Aeronautics and Space Administration Task Load Index; rt-PA: recombinant tissue-plasminogen activator; SUS: system usability scale.

The experiment used a 2-period crossover design to compare the effectiveness of the iTEST with a standard EMR interface. Each user evaluated 4 patients—2 with the iTEST and 2 with the standard EMR—resulting in 48 scenarios (Figure 4A). We set the participant count at 12 to ensure each patient appeared 6 times in both testing scenarios.

The 4 cases chosen for this study are actual patients with stroke from our hospital. They have complex histories, detailed records across multiple departments, and several treatment contraindications. Some critical contraindications are only documented in less prominent sections, like outpatient notes, making them harder to identify quickly during assessment. To prevent overfitting and ensure an unbiased evaluation, the 4 cases were held out from the datasets used for the development or internal validation of the eligibility extraction rules.

At the experiment’s start, each user received a detailed introduction to procedures (Figure 4B) and 10 minutes of iTEST training. A study coordinator managed the process and recorded time points. In period 1, users utilized either the standard EMR interface or the iTEST, switching to the other in period 2. During each period, users evaluated eligibility for 2 patients, first for the rt-PA checklist and then for the milvexian checklist. After evaluations, they assessed cognitive workload using the National Aeronautics and Space Administration Task Load Index (NASA-TLX) scale [19]. At the end of each period, they provided overall usability feedback through the system usability scale (SUS) questionnaire [20].

### Outcomes

The primary outcome measured response accuracy to the eligibility checklist. For each patient, users completed checklists for 2 trials (Tables S2 and S3 in Multimedia Appendix 1). At least 2 senior neurologists (YHS and SFS) with extensive stroke care experience established reference answers by consensus, against which user responses were marked as correct or incorrect. Each correct response earned 1 point, and the final accuracy score (maximum=1) for each checklist was the average of the points of all criteria in that checklist.

Secondary outcomes included checklist completion time, cognitive workload (measured by the NASA-TLX scale), and tool usability (measured by the SUS). The NASA-TLX assesses 2 dimensions of subjective workload: mental demand, physical demand, temporal demand, performance, effort, and frustration [19], rated from 0 to 20, with higher scores indicating greater cognitive workload. Ratings are weighted to produce a total score from 0 to 100. The SUS evaluates interface usability via a 10-item Likert scale questionnaire (from strongly agree to strongly disagree), assessing factors including support needs, training requirements, complexity, integration, ease of learning, and user confidence [20]. SUS scores range from 0 to 100, with higher scores indicating better usability. A prior study showed that adjective ratings of “poor,” “OK,” and “good” corresponded to average SUS scores of 35.7, 50.9, and 71.4, respectively [21].

In addition to the above planned analyses comparing overall performance between the standard EMR interface and the iTEST, we conducted exploratory subgroup analyses examining each treatment protocol (rt-PA and milvexian) separately to provide more detailed insights into system performance across different clinical scenarios.

### Statistical Analysis

Given the small sample size in the user experiment and the non-normal distribution of the outcome measures, we reported the outcome measures as medians with interquartile ranges and conducted nonparametric analyses. The iTEST and the standard EMR interface were compared using the Wilcoxon signed-rank test for repeated user measurements. Two-tailed *P* values <.05 were considered statistically significant. Analyses were performed using R version 4.4.0 (R Foundation for Statistical Computing).

## Results

### User Statistics

Table 1 summarizes participant characteristics. The study included 6 physicians and 6 nurse practitioners. The participants, who were evenly split between male and female participants, had a mean age of 41 years and varied widely in experience levels across clinical practice, EMR use, personal computer use, and stroke care. The median clinical experience was 14.5 years, and they used the standard EMR interface for a median of 7 years.

**Table 1.** User characteristics.

User	Age (y)	Gender	Profession	Experience (y)			
				Clinical practice	EMR^a^ use	PC^b^ use	Stroke care
#1	34	Male	Physician	7	5	7	1
#2	46	Female	NP^c^	25	15	30	5
#3	51	Female	NP	27	9	30	16
#4	39	Female	NP	10	10	20	16
#5	40	Female	NP	20	8	20	3
#6	32	Male	Physician	8	7	15	1
#7	40	Male	Physician	14	6	25	6
#8	35	Male	Physician	10	3	25	3
#9	41	Male	Physician	15	1	22	15
#10	43	Male	Physician	16	12	20	16
#11	40	Female	NP	18	7	22	2
#12	48	Female	NP	7	7	7	3

a EMR: electronic medical record.

b PC: personal computer.

c NP: nurse practitioner.

### Computational Performance

To evaluate the iTEST’s computational performance, we measured processing time using EMRs from 15 patients with different documentation volumes. The number of clinical notes per patient ranged from 417 to 7299. System processing time (measured from patient selection to the full display of MetaMap Lite-analyzed clinical notes) varied from 6 to 345 seconds (average: 39 ms per note). This processing represents an initial computational overhead when a patient is first loaded; the subsequent evaluation of multiple eligibility criteria requires no extra processing time. In our clinical workflow, iTEST processing begins at patient triage in the emergency department, ensuring that all EMR analyses are completed before clinicians assess trial eligibility.

### Evaluation Outcomes

Table 2 compares the outcomes of the standard EMR interface with the iTEST. Users attained a median accuracy score of 0.91 for the primary outcome using the standard EMR interface. In contrast, the iTEST produced a higher median accuracy score of 1.00, representing a 9.9% improvement with strong statistical significance (*P*<.001).

**Table 2.** Comparisons of outcomes between scenarios using the standard EMR^a^ interface and the iTEST.

Outcome	Standard EMR interface, median (IQR)	iTEST^b^, median (IQR)	*P* value
Accuracy score	0.91 (0.84‐0.96)	1.00 (0.95‐1.00)	<.001
Time (min)	3.18 (2.34‐4.52)	2.44 (1.67‐3.17)	.004
NASA-TLX^c^ score	62.8 (36.7‐75.8)	39.7 (26.8‐52.0)	.02
SUS^d^ score	46.3 (36.9‐55.0)	71.3 (56.9‐75.6)	.01

a EMR: electronic medical record.

b iTEST: intelligent trial eligibility screening tool.

c NASA-TLX: National Aeronautics and Space Administration Task Load Index.

d SUS: system usability scale.

Tables S2 and S3 in Multimedia Appendix 1 display the average accuracy scores of all 12 users for each eligibility criterion. With the standard EMR interface, accuracy scores ranged from 0.54 to 1.00, while the iTEST improved this range to 0.75 to 1.00. Users particularly struggled with certain criteria while using the standard interface, including identifying a diabetes history along with a prior stroke, internal bleeding within the last 3 months, and an activated prothrombin time of ≤1.4 times the control value within 7 days. For these challenging criteria, accuracy scores dropped below 0.60 with the standard interface but improved to at least 0.75 with the iTEST.

For secondary outcomes, the iTEST reduced task completion time by 0.74 minutes (23.3% reduction; from 3.18 to 2.44 min; *P*=.004) compared with the standard EMR interface. Users reported a 23.1-point reduction in NASA-TLX scores (36.8% decrease; from 62.8 to 39.7; *P*=.02), indicating substantial improvement in perceived cognitive demand. The SUS scores improved by 25 points (54% increase; from 46.3 to 71.3; *P*=.01), moving the system from the “POOR to OK” category to the “OK to GOOD” category and exceeding the acceptability threshold [21].

Table 3 presents the scores for each NASA-TLX subscale. Among the 6 dimensions of cognitive workload, mental demand, temporal demand, and effort received higher scores than physical demand, performance, and frustration. Participants reported significant reductions in temporal demand, effort, and frustration when using the iTEST as opposed to the standard EMR interface.

**Table 3.** Comparisons of NASA-TLX^a^ subscales between scenarios using the standard EMR^b^ interface and the iTEST^c^.

NASA-TLX subscale	Standard EMR interface, median (IQR)	iTEST, median (IQR)	*P* value
Mental demand	13.5 (10.0‐16.6)	10.0 (6.8‐14.3)	.13
Physical demand	10.0 (6.8‐13.0)	7.0 (4.4‐10.0)	.18
Temporal demand	14.0 (10.0‐17.6)	9.0 (4.9‐11.5)	.007
Performance	7.0 (2.0‐10.5)	3.8 (2.0‐5.3)	.10
Effort	14.0 (10.0‐17.6)	9.0 (4.4‐14.0)	.02
Frustration	7.5 (3.5‐10.0)	4.3 (1.0‐8.0)	.02

a NASA-TLX: National Aeronautics and Space Administration Task Load Index.

b EMR: electronic medical record.

c iTEST: intelligent trial eligibility screening tool.

The results of the exploratory subgroup analysis, which compares the outcomes for each checklist individually, are listed in Table S4 in Multimedia Appendix 1. For the rt-PA checklist, the accuracy score significantly improved from 0.91 to 1.00 (*P*=.001) using the iTEST. However, no significant differences were observed in task completion time or NASA-TLX scores. In contrast, the iTEST significantly improved all outcomes for the milvexian checklist.

## Discussion

### Principal Results

This study demonstrated that the iTEST outperformed the standard EMR interface in multiple ways. It improved the accuracy of determining trial eligibility criteria while enhancing efficiency through shorter task completion time. The iTEST also significantly reduced users’ cognitive workload and offered better usability than the standard interface.

### Accuracy of Eligibility Screening

The iTEST showed significant improvements over the standard EMR interface. It enhanced accuracy in identifying both numerical laboratory data and textual information in clinical documents. For instance, for the milvexian checklist, the accuracy score for “APTT≤1.4 times control” increased markedly from 0.54 to 0.92 (Table S3 in Multimedia Appendix 1). This improvement likely occurred because the standard EMR lacks automated rule checking, requiring users to perform mental or manual calculations of numerical values.

The iTEST also improved accuracy when determining eligibility criteria using textual information. For instance, for the rt-PA checklist, users often misidentified patients with “diabetes with prior stroke,” achieving only a 0.54 accuracy score. This low score likely arose from the complexity of the assessments needing both laboratory data and clinical narratives. Extensive medical histories can obscure crucial diagnostic evidence, leading to medical errors [22]. This is a common challenge for clinical researchers working with large, complex medical records—even in electronic form, the human brain struggles to identify key concepts quickly [23,24]. The iTEST addressed this challenge by extracting and highlighting essential information in clinical documents, thereby improving eligibility screening accuracy from 0.54 to 0.75 (Table S2 in Multimedia Appendix 1). However, the suboptimal accuracy (0.75) underscores the challenge of linking temporal relationships between conditions documented across multiple clinical notes. This limitation affects both automated systems and human reviewers due to cognitive load and fragmented information in medical records.

While the iTEST performed well overall in clinical trial screening, our NLP validation (Table S1 in Multimedia Appendix 1) revealed issues with key concept extraction. Notably, “Anticoagulant” detection (F_1_-score=0.33) was poor, mainly due to nonstandard negation expressions that caused false positives and incomplete concept mapping, leading to missing specific anticoagulants. We refined the process by adding 5 anticoagulants (warfarin, apixaban, dabigatran, edoxaban, rivaroxaban), greatly improving accuracy. This workaround, however, highlights a significant limitation of MetaMap Lite’s concept generalization and ontology depth for broad drug classes; the tool failed to effectively handle a critical, broad safety concept without explicit enumeration of individual drugs. This highlights the need for concept validation and iterative refinement in NLP tools for critical criteria.

Furthermore, using MetaMap Lite instead of the full MetaMap may result in less effective concept extraction. While faster and lighter, MetaMap Lite implements only a subset of MetaMap’s extensive options and lacks sophisticated word-sense disambiguation modules, which can result in mapping all available senses of a term and generating false positives [17]. The failure to generalize the anticoagulant concept exemplifies this limitation. Specifically, MetaMap Lite’s shallower ontology proved inadequate for managing hierarchical drug class relationships essential for safety screening. Although suitable for resource-limited or real-time applications, high-accuracy tasks are better served by the full MetaMap, emphasizing the trade-off between speed and depth.

Therefore, we recommend that clinical implementations of the iTEST include a validation phase where each new eligibility criterion is tested against a small set of annotated medical records before deployment. This quality assurance step can identify concept-mapping issues and help refine the process, especially for critical safety−related criteria such as anticoagulant use. Furthermore, despite the iTEST achieving a median accuracy of 1.00, the relatively lower accuracy on critical, complex criteria such as “diabetes with prior stroke” still indicates a high-risk failure mode that the iTEST did not fully address.

### Efficiency and Usability

Even though users were trying the iTEST for the first time, they completed eligibility screening significantly faster after just a 10-minute training session. Users took a median of 2.44 minutes with the iTEST compared to 3.18 minutes with the standard EMR interface (Table 2) despite having years of experience with the latter. Notably, when analyzing individual checklists, the rt-PA checklist showed no significant improvement in completion time. This likely occurred because users were already highly familiar with checking eligibility criteria for intravenous thrombolysis (similar to those of the NINDS rt-PA trial) in their daily clinical practice before the study. Additionally, our study might have been underpowered to detect these smaller effects on familiar tasks, unlike the significant improvements seen across all metrics for the unfamiliar milvexian checklist.

With users achieving higher accuracy in less time during eligibility screening, it is unsurprising that the iTEST demonstrated better system usability than the standard EMR interface. The SUS score of 71.3 represents acceptable usability in the “OK to GOOD” range, indicating meaningful improvement over the standard EMR (46.3) but also suggesting room for further optimization. Despite being new to users and having received only 10 minutes of training, the iTEST demonstrated reasonable ease of use for this specific task. Research has shown that EMR systems should incorporate ongoing physician feedback to enhance usability, as poor EMR design can negatively impact physicians’ well-being and increase their perceived workload [25]. Consistent with these findings, the iTEST not only improved system usability but also reduced the perceived cognitive workload.

### Cognitive Workload

The iTEST significantly outperformed the standard EMR interface in overall NASA-TLX scores. While some researchers question the mathematical validity of combining the 6 NASA-TLX dimensions into a single workload score [26], we analyzed both the overall and individual dimensions (Table 3). The most substantial improvements were observed in temporal demand (from 14.0 to 9.0, *P*=.007) and effort (from 14.0 to 9.0, *P*=.02), with frustration levels also showing a significant reduction (from 7.5 to 4.3, *P*=.02). The marked reduction in temporal demand aligned with the decreased task completion time, which is crucial since the effectiveness of stroke therapy depends heavily on timing [27] and creates substantial pressure for clinicians [28,29]. While we observed significant improvements in temporal demand, effort, and frustration subscales, reductions in mental demand, physical demand, and performance subscales did not reach statistical significance, possibly due to a limited sample size.

Few studies have assessed trial eligibility screening workload [30,31], primarily measuring work volume instead of cognitive burden. To our knowledge, no research has specifically analyzed the cognitive workload of this task. However, studies have compared perceived cognitive workload across different EMR interfaces and visualization tools for various clinical tasks [32-34], indicating that renovations to the EMR interface can enhance efficiency and reduce cognitive demands.

### Clinical Implications

The iTEST improves upon the standard EMR interface with better accuracy and efficiency in eligibility screening, reduced cognitive workload, and enhanced usability, leading to several clinical benefits. Researchers can enroll suitable trial participants more effectively, ensuring scientific validity and data quality while protecting ineligible patients. Additionally, the iTEST enhances patient safety by avoiding unnecessary risks. For instance, the NINDS-rt-PA trial, which examined rt-PA in patients with acute ischemic stroke, highlights the importance of careful patient selection to mitigate serious intracranial bleeding risks [35]. The eligibility criteria established by this trial have become the standard for intravenous thrombolysis [36]. Consequently, the iTEST can assist clinicians in selecting appropriate patients for therapies, improving routine clinical practice.

While the iTEST’s computational overhead can reach up to 345 seconds for patients with extensive EMR histories, this does not affect time-sensitive clinical decision-making in our workflow. By starting EMR processing at emergency department triage, all computational work is completed before clinicians evaluate trial eligibility. This integration ensures that the iTEST’s processing time does not cause delays during the critical acute stroke assessment window. Future implementations in other clinical settings should consider similar workflow integration strategies, and improving MetaMap Lite’s performance may further reduce processing times for real-time use.

This study examined 2 checklists. Users were already familiar with the rt-PA checklist before the study. They still made occasional mistakes, achieving a median accuracy score of 0.91. The iTEST improved this score to 1.00. The milvexian checklist, being new to users, yielded a lower median accuracy score (0.84) than the rt-PA checklist (0.91), highlighting how unfamiliarity with eligibility criteria can lead to more errors. With the iTEST, users achieved nearly perfect accuracy (median score 1.00) for both the familiar rt-PA and unfamiliar milvexian checklist, demonstrating its value over the standard EMR interface, especially for new eligibility checklists. However, the separate analyses of the rt-PA and milvexian checklists were exploratory. While these findings provide valuable insights into protocol-specific performance, they should be interpreted cautiously and confirmed in future prospective studies with predefined hypotheses.

Cognitive overload is a leading cause of burnout among clinicians [4]. When facing burnout, clinicians favor patients with straightforward medical histories, as fewer clinical documents are needed for eligibility determination. They may overlook patients with complex, lengthy medical histories requiring thorough review. This selection bias can compromise clinical trial validity by causing the trial population to deviate from the intended target population. The iTEST addresses this issue by reducing cognitive workload, thus streamlining patient recruitment and minimizing selection bias. This benefit becomes even more pronounced as EMR documentation grows over time.

Prior research on trial eligibility screening has primarily focused on enhancing recruitment efficiency through the application of NLP and machine learning to EMR data. The foundational work by Ni et al [30,31] demonstrated substantial workload reductions, with over 90% in pediatric emergency department settings and 85% in pediatric oncology, by leveraging both structured data and unstructured clinical notes. The subsequent development of the Automated Clinical Trial Eligibility Screener [9] advanced this work into real-time, prospective screening, achieving a 34% reduction in screening time and an SUS score of 80.0.

The iTEST extends this foundation by addressing clinician empowerment and cognitive burden through 2 key innovations. First, its block-based visual programming interface enables clinicians to independently author and update screening rules without software engineering support, enhancing adaptability to evolving eligibility criteria. Second, the iTEST demonstrated significant improvements not only in efficiency but also in accuracy and cognitive workload, quantitatively validating its ability to reduce mental burden while maintaining acceptable usability. This positions the iTEST as a clinician-centric solution that enhances both the efficiency and reliability of eligibility screening while reducing clinician burnout.

### Future Direction

Clinical notes at our hospital are primarily in English, with very few in Chinese. Therefore, we disregarded the Chinese portions when developing the iTEST. Besides, extracting medical concepts from non-English clinical documents presents significant challenges [37]. However, advancements in large language models (LLMs) may help address the challenge of multilingual clinical notes. They could potentially process multilingual clinical notes without explicit translation, reducing information loss. Moreover, LLMs provide promising capabilities that could overcome several limitations of rule-based approaches, such as those used in our study. Their natural language understanding could improve the handling of variations in clinical documentation, context-dependent interpretations, and complex eligibility criteria that require reasoning across multiple data points.

However, several issues must be addressed before widespread clinical use. First, reliability and consistency remain major challenges [38,39]. LLMs can produce inconsistent outputs for the same inputs and may generate plausible but incorrect information (“hallucinations”), which is unacceptable in clinical decisions. Second, computing costs and infrastructure requirements could limit scalability compared to rule-based systems. Third, regulatory compliance presents significant obstacles, as current frameworks require explainable, auditable decision-making processes, but LLM reasoning often lacks transparency. Finally, concerns about data privacy and the need for specialized medical training data require careful consideration [40].

Future hybrid approaches that combine rule-based reliability with LLM flexibility may offer the best solutions [41], where LLMs handle complex language understanding and concept extraction [42]. Conversely, rule-based systems guarantee consistent application of explicit eligibility criteria. Importantly, such hybrid frameworks can improve explainability by maintaining transparent decision pathways: the rule-based component offers clear, traceable logic for eligibility determinations, while LLM outputs can be limited to produce interpretable explanations for their reasoning processes. In this way, LLMs, with their strong reasoning and summarization abilities, could be useful in various clinical and research scenarios [43].

### Limitations

This study has several limitations. First, with only 12 clinicians evaluating 4 patients’ eligibility, the study findings may not reflect real-world scenarios. Furthermore, the small number of participants may lack enough statistical power to detect differences between the groups. Although our study design, with 12 clinicians, was appropriate for identifying medium-to-large differences, we acknowledge that it may have been underpowered to detect smaller, yet potentially clinically important, effects. Despite this limitation, all 4 outcomes achieved statistical significance. Second, the 4 patients chosen for this study may not accurately represent a typical patient population. However, these cases truly demonstrate the difficulties clinicians encounter when trying to retrieve crucial information from traditional EMR systems. In real-world clinical settings, these patients represent a specific group that not only risks misjudgment and possible harm but also presents challenges for clinical trials.

Third, while our crossover design enhances internal validity through within-subject comparisons, its applicability to real-world recruitment is limited due to the small number of participants and patient scenarios. These may not accurately reflect the diversity of clinical experience, patient complexity, practice patterns, or technological skills across different settings. Real-world implementation involves a wider range of patient presentations, comorbidities, and situations that could impact system performance differently. Future research should test the iTEST in diverse clinical environments with larger samples to better determine its effectiveness for large-scale trial recruitment. Fourth, the controlled experimental setting may not fully capture the time pressures, interruptions, and multitasking demands of actual emergency care that could influence accuracy and efficiency.

Fifth, the brief 10-minute training and the crossover design could theoretically lead to learning effects [44] that may influence our results. Although we counterbalanced the task order to minimize these effects, the crossover design means participants’ second interface evaluation might have been affected by familiarity with the eligibility screening task itself. However, the counterbalancing of task order should have evenly distributed any such learning effects across conditions [45]. Additionally, the short 10-minute training period for the iTEST actually serves as a conservative test of the system’s usability. The fact that the iTEST showed superiority despite minimal training strengthens rather than weakens our findings.

Sixth, we acknowledge that our user study focused exclusively on the rule evaluation module and did not assess clinicians’ ability to author rules using the block-based interface. Our study design prioritized evaluating whether the system could effectively support the eligibility screening workflow with preconfigured rules, as this represents the most common use case in our target clinical settings. However, we recognize that this does not validate the authoring capabilities.

Finally, while participants had extensive experience with the standard EMR interface compared to minimal training with the iTEST, we cannot rule out the influence of the novelty effect [46] or the Hawthorne [47] effect on our results. Participants’ awareness of being observed and evaluated, along with the novelty of the iTEST interface, may have led to increased attention and improved performance. Future studies with longer training periods, repeated testing, and naturalistic observation could help determine whether the observed benefits persist beyond initial exposure, revealing learning curves for both systems.

### Conclusions

In this study, we introduced the iTEST, an innovative NLP-powered clinical decision support tool that uses MetaMap Lite to extract relevant concepts from EMRs. The system design includes a block-based visual programming interface intended to enable clinicians to author and modify eligibility rules independently; however, the usability and effectiveness of this authoring capability were not evaluated in this study and remain objectives for future research. Our user evaluation focused exclusively on the rule evaluation module using preconfigured rules, where the iTEST demonstrated superior accuracy and usability compared to the standard EMR interface, while reducing both task completion time and perceived cognitive workload. The tool’s applications can extend beyond clinical trial screening to include the verification of indications and contraindications for specific therapies or interventions. Through these capabilities, the iTEST has the potential to enhance patient safety while easing clinicians’ cognitive burden. Future research should evaluate clinicians’ ability to create and modify eligibility rules using the block-based authoring interface to fully validate the system’s empowerment objectives and finalize the assessment of the iTEST’s intended functionality.

## Supplementary material

10.2196/80072 Multimedia Appendix 1 Supplementary Tables S1–S4 showing additional study results.

## Abbreviations

iTEST: intelligent trial eligibility screening tool
LLM: large language model
NASA-TLX: National Aeronautics and Space Administration Task Load Index
NINDS: National Institute of Neurological Disorders and Stroke
EMR: electronic medical record
NLP: natural language processing
rt-PA: recombinant tissue-plasminogen activator
SUS: system usability scale
