# Supplementary material for: An Intelligent Trial Eligibility Screening Tool Using Natural Language Processing With a Block-Based Visual Programming Interface: Development and Usability Study
Source: JMIR Med Inform. 2025 Dec 11;13:e80072. doi: 10.2196/80072 (PMC12698033; doi:10.2196/80072)
Supplement: Multimedia Appendix 1 [file medinform-v13-e80072-s001.pdf]

Multimedia Appendix 1: Supplementary Tables

Table S1. Performance of identifying medical concepts using MetaMap Lite.

Concept	CUI[a]	Precision	Recall	F1
Acute cerebrovascular accidents	C0751956	1.00	1.00	1.00
Acute infarct	C0333548	1.00	1.00	1.00
Acute ischemic stroke	C5392833	1.00	1.00	1.00
Anticoagulant	C3536711	0.33	0.33	0.33
Apixaban	C1831808	1.00	1.00	1.00
Brain neoplasms	C0006118	1.00	1.00	1.00
Cavernous hemangioma of brain	C2919945	1.00	1.00	1.00
Cerebral infarction	C0007785	0.71	0.71	0.71
Cerebrovascular accident	C0038454	0.66	0.63	0.64
Cerebrovascular disorders	C0007820	1.00	1.00	1.00
Chronic kidney disease stage 5	C2316810	1.00	1.00	1.00
Congestive heart failure	C0018802	1.00	1.00	1.00
Coumadin	C0699129	1.00	1.00	1.00
Craniocerebral trauma	C0018674	1.00	1.00	1.00
Diabetes	C0011847	1.00	1.00	1.00
Diabetes mellitus	C0011849	0.97	0.92	0.94
Diskectomy	C0206078	1.00	1.00	1.00
Duodenal ulcer	C0013295	1.00	1.00	1.00
Edoxaban	C2975435	1.00	1.00	1.00
Epilepsies, partial	C0014547	1.00	1.00	1.00
Epilepsy	C0014544	1.00	1.00	1.00
Gastrointestinal hemorrhage	C0017181	1.00	1.00	1.00
Heparin, porcine	C0770546	1.00	1.00	1.00
Hepatic insufficiency	C1306571	1.00	1.00	1.00
Hepatitis	C0019158	1.00	1.00	1.00
Infarction	C0021308	0.78	0.75	0.76
Infarction, lacunar	C0333559	0.83	0.63	0.71
International normalized ratio	C0525032	1.00	1.00	1.00
Intracranial hematoma	C0596793	1.00	1.00	1.00
Ischemic stroke	C0948008	0.80	0.80	0.80
Kidney failure, chronic	C0022661	1.00	1.00	1.00
Liver cirrhosis	C0023890	1.00	1.00	1.00
Liver failure	C0085605	1.00	1.00	1.00
Metastatic malignant neoplasm to brain	C0220650	1.00	1.00	1.00
Operation on brain	C0195775	1.00	1.00	1.00
Peptic ulcer	C0030920	1.00	1.00	1.00
Pradaxa	C2940579	1.00	1.00	1.00
Rivaroxaban	C1739768	1.00	1.00	1.00
Seizures	C0036572	1.00	1.00	1.00
Seizures, focal	C0751495	1.00	1.00	1.00

Subarachnoid hemorrhage	C0038525	1.00	1.00	1.00

[a]CUI: concept unique identifier.

Table S2. Average accuracy scores of all users for each eligibility criterion in the rt-PA checklist.

Time frame	Eligibility criterion for rt-PA[a]	Accuracy score	
		Standard EMR[b] interface	Trial eligibility screening tool
Within 7 d	Platelets <100,000/mm^3	0.88	0.96
Within 3 d	Blood glucose <50 or >400 mg/dL	0.92	1.00
History	Cerebral hemorrhage	1.00	0.92
	Any aneurysm	0.83	0.88
	Arteriovenous malformation	1.00	1.00
	Brain surgery history	1.00	1.00
	Spinal surgery history	1.00	1.00
Within 3 m	Head trauma	1.00	0.96
History	Liver failure, cirrhosis, esophageal varices/bleeding	1.00	1.00
Within 7 d	Acute hepatitis (liver index more than 3 times normal)	0.96	1.00
History	Infective endocarditis, pericarditis, history of aortic dissection	0.92	1.00
Within 3 m	Gastrointestinal bleeding, active internal bleeding	0.79	0.96
History	Hemorrhagic eye diseases	0.75	0.96
	Heart failure	0.75	0.96
Within 1 m	Acute pancreatitis	1.00	1.00
Within 7 d	Childbirth	1.00	1.00
Within 3 m	Use of anticoagulants	0.92	0.96
History	Diabetes with prior stroke	0.54	0.75
	Intracranial tumor	1.00	1.00
	Dialysis	0.96	1.00
Within 7 d	INR[c] >1.3	1.00	1.00
	APTT[d] >2 times control	0.96	1.00
Within 2 d	Use of heparin	1.00	1.00

[a]rt-PA: recombinant tissue-plasminogen activator.
[b]EMR: electronic medical record.
[c]INR: international normalized ratio.
[d]APTT: activated partial thromboplastin time.

Table S3. Average accuracy scores of all users for each eligibility criterion in the milvexian checklist.

Time frame	Eligibility criteria for milvexian	Accuracy score	
		Standard EMR[a] interface	Trial eligibility screening tool
Within 7 d	INR[b] ≤1.5	0.63	0.92
	APTT[c] ≤1.4 times control	0.54	0.92
History	History of intracranial hemorrhage	1.00	1.00
Within 3 m	Use of anticoagulants	0.83	0.92
	Internal bleeding	0.58	0.83
History	Spinal bleeding	1.00	1.00
	Head trauma	1.00	0.96
	Retinal hemorrhage	0.75	0.96
Within 1 m	Acute hepatitis	0.92	1.00
Within 7 d	ALT[d] >3 times normal	0.96	0.96
History	Cirrhosis	0.92	1.00
	Dialysis	0.96	1.00
Within 7 d	eGFR[e] <15	0.92	1.00
	Platelets <75,000/mm^3	1.00	1.00
	Total bilirubin ≥ 1.5 times normal	0.88	1.00
	Hb[f] <8	1.00	1.00
	INR >1.5	1.00	1.00
	APTT >1.4	0.96	1.00
History	Bleeding tendency	0.67	0.88

[a]EMR: electronic medical record.
[b]INR: international normalized ratio.
[c]APTT: activated partial thromboplastin time.
[d]ALT: alanine aminotransferase.
[e]eGFR: estimated glomerular filtration rate.
[f]Hb: hemoglobin.

Table S4. Comparisons of outcomes between scenarios using the standard EMR interface and the iTEST.[a]

	Standard EMR[b] interface	iTEST[c]	*P*
rt-PA[d] checklist			
Accuracy score	0.91 (0.90–0.96)	1.00 (0.95–1.00)	.001
Time, minute	3.37 (2.94–4.87)	2.75 (1.68–4.29)	.10
NASA-TLX[e] score	62.8 (36.7–75.7)	42.5 (30.3–54.3)	.25
Milvexian checklist			
Accuracy score	0.84 (0.79–0.95)	1.00 (0.95–1.00)	<.001
Time, minute	2.57 (2.04–3.91)	2.03 (1.67–2.88)	.02
NASA-TLX score	65.2 (42.7–76.2)	36.2 (20.1–46.0)	.03

[a]Data are given as median (interquartile range).
[b]EMR: electronic medical record.
[c]iTEST: intelligent trial eligibility screening tool.
[d]rt-PA: recombinant tissue-plasminogen activator.
[e]NASA-TLX: National Aeronautics and Space Administration Task Load Index.
